# Sublingual epidermoid cyst: a case report

**DOI:** 10.4076/1757-1626-2-8848

**Published:** 2009-09-09

**Authors:** Karthikeya Patil, VG Mahima, Suchetha N Malleshi

**Affiliations:** 1 Department of Oral Medicine and Radiology, JSS Dental College and Hospital, SS Nagar, Mysore, India - 570015

## Abstract

Of all the epidermoid cysts encountered throughout the body, only 7% occurs in the head and neck area, with the oral cavity accounting for only 1.6%. Intraorally this benign slow growing and painless entity is usually located in the submandibular, sublingual and submental region. They can cause symptoms of dysphagia and dyspnoea and have a malignant transformation potential. Surgical excision is the treatment of choice. Described here is a case of gigantic sublingual epidermoid cyst.

## Introduction

Epidermoid cysts are benign pathologies that can occur anywhere in the body, predominantly seen in areas where embryonic elements fuse together.^1^ Most cases have been reported in the ovaries and the testicles (80%), with head and neck accounting for 7% [1,2]. Dermoid and epidermoid cysts in the mouth are uncommon and comprise less than 0.01% of all the oral cysts [2-4]. Majority of them occur in sublingual region, but there are rare case reports of occurrence in other sites.

## Case presentation

A 28-year-old male of Indian origin, who was moderately built and nourished presented for treatment of mobile lower front teeth. However, the patient had also noticed a swelling in the floor of the mouth of five months duration. Initially pea sized, the swelling had constantly and gradually increased in size. The swelling nevertheless did not cause any pain, discomfort, dysphagia nor speech or masticatory difficulties to the patient.

**Figure 1:**
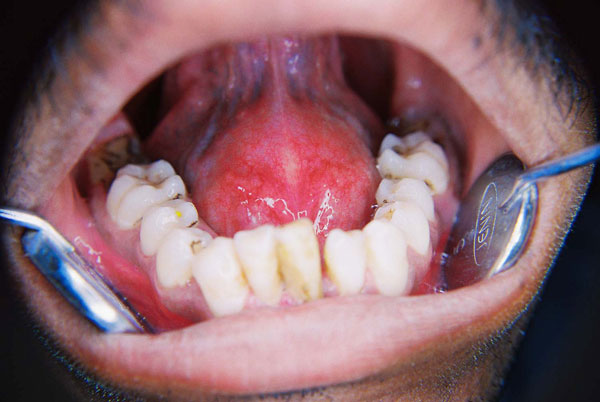
**Pre operative photo showing dome shaped sublingual swelling**.

Extra orally there was no clue of the swelling. Clinical intra oral examination revealed periodontally compromised mandibular anteriors. The floor of the mouth revealed a solitary, well circumscribed, distinct, dome shaped sessile midline swelling extending from the lingual aspect of the mucogingival junction of mandibular anterior teeth up to the mandibular molars bilaterally. The mucosa over the swelling appeared normal without any secondary changes. Tongue was slightly raised but the morphology of the swelling did not vary with tongue moment (Figure 1). On palpation the swelling was soft to firm, non tender, smooth, fluctuant and was not associated with any discharge. Although submandibular and sublingual gland orifices could not be assessed because they were masked by the swelling, bilateral milking of the glands produced thick, mucous saliva.

Mandibular cross sectional radiograph did not disclose any calcifications. The buccal and lingual cortical plates were normal with no indication of expansion or decortication.

Aspiration from two different sites using an 18 gauge needle yielded scanty white cheesy material. Complete hemogram and ESR were within normal limits.

Ultrasonography showed a well defined submucosal oval mass in floor of mouth in the midline measuring approximately 4.0 × 3.0 cms in diameter with internal echoes. The underlying tongue musculature was normal.

FNAC showed few squamous cells with mild hyperchromatic nuclei visible in the background of sheets of macrophages, few lymphocytes, abundant keratin flakes and anucleate squames.

**Figure 2:**
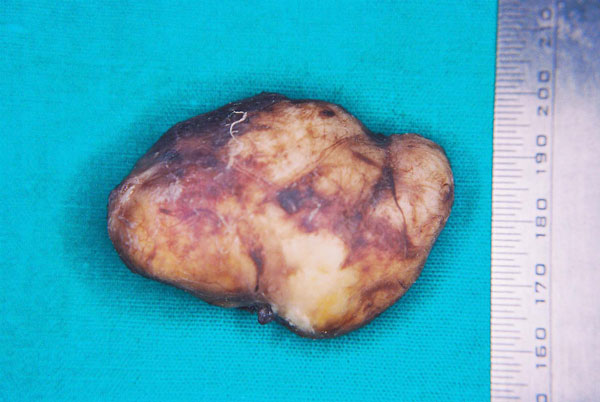
**Photograph of excised cyst**.

**Figure 3:**
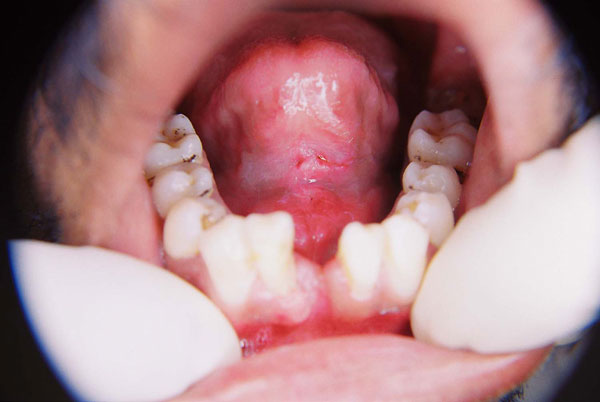
**Post operative photograph**.

Excision of the swelling under local anesthesia yielded a yellowish white smooth surfaced oval mass of tissue measuring approximately 3 × 2 × 2 cms which was soft in consistency and cystic in nature (Figure 2). The mass upon sectioning was filled with a cheesy material. Post operative healing period was uneventful (Figure 3).

**Figure 4:**
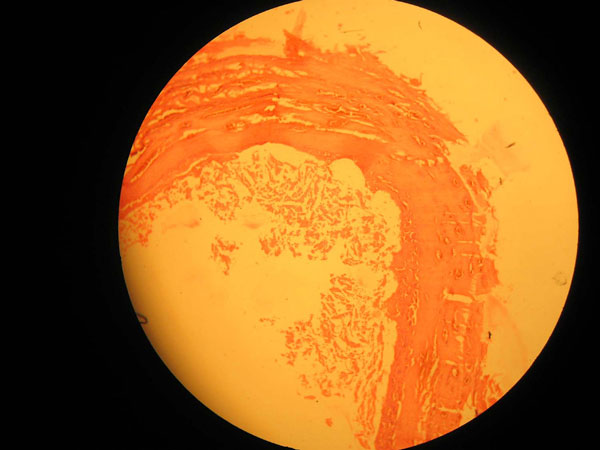
**Low power photomicrograph showing cystic cavity with keratin flakes**.

**Figure 5:**
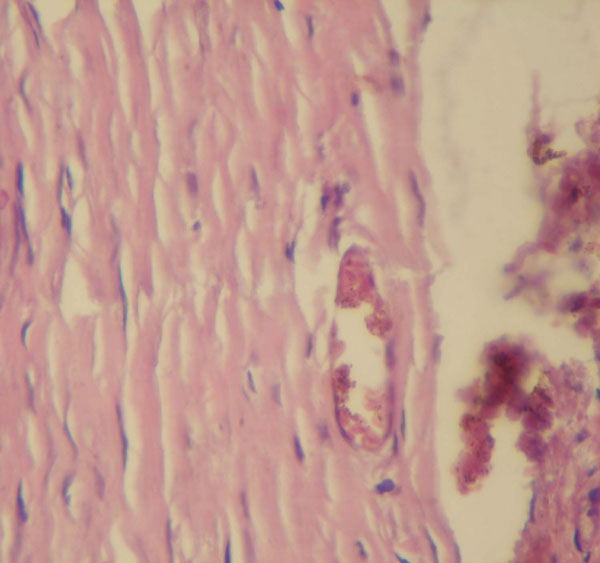
**High power view of keratin lining cystic cavity**.

The histopathologic investigation showed cystic cavity filled with keratin flakes with the epithelial lining discontinuous and missing in certain areas. Connective tissue showed lymphocytes and multinucleated giant cells with deeper tissue fibrosis and prominent blood vessels (Figures 4 and 5). Hence, it was conclusively diagnosed as epidermoid cyst.

## Discussion

Epidermoid and dermoid cysts are rare, benign lesions found throughout the body, with 7% occurring in the head and neck area, 1.6% of which occurs in the oral cavity. Of all the oral cysts dermoid cysts account for only 0.01% [2-4] Roser was the first to designate dermoid cysts in the floor of the mouth as epidermoid tumours [5].

Even though the expression "dermoid cyst" characterizes a distinct entity, the word "dermoid" has been used to designate true dermoid cysts, epidermoid cysts, and teratoid cysts [2,3,6].

Based on the histopathological picture Meyer divided the floor of the mouth cysts into following types: [1,2,4].

1.Â Epidermoid cysts - where in the cystic cavity is lined with epithelium without skin appendages.

2.Â Dermoid cysts - here the epithelial lined cystic cavity encloses skin appendages such as hair, hair follicles, sebaceous, and sweat glands.

3.Â Teratoid cysts - in this entity, the cystic cavity in addition to skin appendages also encloses mesodermal derivatives such as bone, muscle, gastrointestinal and respiratory tissue.

All these three cysts owing to their squamous epithelium lining may enclose cheesy keratinaceous material within their lumen. Hence the fundamental difference between the dermoid and the epidermoid is the presence of skin appendages within the wall of the former and the lack of the same in the latter [3,6].

Epidermoid cysts may be categorized as congenital or acquired based on their origin although there is no disparity between the two either clinically or histologically [2,4,7].

Ambiguity about their exact pathogenesis exists and dysontogenetic, traumatic, and thyroglossal anomaly theories have been postulated [1,2,4,6]. Most congenital dermoid and epidermoid cysts perhaps begin due to an embryologic accident during the early stages of development but hardly get perceived until their size causes annoyance [3]. The origin of epidermoid cysts is believed to be from entrapment of epithelial remnants during midline closure of the bilateral first and second branchial arches [1,4,5,7]. It has also been opined that ectodermal differentiation of multipotential cells, most probably pinched off at the point of anterior neuropore closure may give rise to these cysts [3]. On the other hand, they may also crop up from the tuberculum impar of His [4,5].

Traumatic or iatrogenic inclusion of epithelial cells or the blockage of a sebaceous gland duct have been postulated as the pathogenesis of acquired cyst [4,7]. However, some authors have also stated that midline cysts may represent a diverse form of thyroglossal duct cyst [4,6,7].

They may be found in any age group but show preponderance between 15-35 years of age with no gender predilection [1,4,5]. Although floor of the mouth in the midline is most favored site, occasional occurrence involving the buccal mucosa, tongue, lips, uvula, temporomandibular joint dermal graft, intradiploic, intracranial, and intraosseous location within the mandible and maxilla also have been cited in literature [4,8,9]. These lesions show variation in size and weight from few millimeters to centimeters and a gram to several hundred gram respectively [3,5]. Symptoms of dysphagia, dyspnoea and dysphonia may occur due to upward displacement of tongue by these sublingual swellings [4,5]. Further more growth in a inferior direction may give rise to appearance of characteristic "double chin" [1,4,8]. These well encapsulated lesions typically feel "dough like" on palpation, although they may be fluctuant and cyst like based on consistency of the luminal contents, that may range from a cheesy, sebaceous to liquefied substance [1,5,6].

Fine needle aspiration cytology, ultrasound, CT and MR imaging provide essential information on the cyst location that allows optimal preoperative planning. Ultrasonographic findings comprise solid and cystic structures within a heterogeneous mass [3]. On CT scans, the dermoids appear as moderately thin walled, unilocular masses filled with a homogeneous, hypoattenuating fluid substance with numerous hypoattenuating fat nodules giving the pathognomonic "sack-of-marbles" appearance [3]. On MR imaging dermoid cysts give variable signal intensity on T1-weighted images and are usually hyperintense on T2-weighted images [3,9]. Fine needle aspiration cytology has been advocated as an essential investigation. Although not equivalent to CT and MRI, it is safe, economical and dependable technique and is therefore useful for analysis of sublingual lesions [1,6].

The differential diagnosis for sublingual dermoids should comprise ranula, unilateral or bilateral blockage of Wharton's ducts, lipoma, thyroglossal duct cyst, cystic hygroma, branchial cleft cysts, acute infection or cellulitis of the floor of the mouth, infections of submaxillary and sublingual salivary glands, floor of the mouth and adjacent salivary glands benign and malignant tumors, heterotopic gastrointestinal cyst and duplication foregut cyst [1,2,4,6].

Treatment comprises total surgical excision [1,2,4]-[6]. Caution should be taken not to rupture the cyst, as cystic contents may act as irritants to fibrovascular tissues, causing postoperative inflammation [3]. Recurrences are unusual after absolute surgical excision [1,3]. Reports of malignant transformation of sublingual dermoid and epidermoid to squamous carcinoma and basal cell carcinoma are present [1,2]. A 5% rate of malignant transformation of the teratoid variety of oral dermoid cysts has also been quoted in literature [3]-[5].

## Conclusion

Epidermoid cyst of the oral cavity is an uncommon entity. Ample understanding and vigilance about this slow growing painless mass is essential not only because of the symptoms it produces but also due to its malignant potential.

## Consent

All reasonable attempts to obtain patient's permission/consent have been made, however the authors opine that there is no reason to think that the patient or their family would object to publication. The identity of the patient has not been revealed either in the text or in the photographs.

## Competing interests

The authors declare that they have no competing interests.

## Authors' contributions

All the authors have contributed significantly towards the preparation of the final manuscript.
